# The impact of drone delivery of an automated external defibrillator: a simulation feasibility study

**DOI:** 10.29045/14784726.2025.6.10.1.56

**Published:** 2025-06-01

**Authors:** Owen Finney, Kate Snowdon, Sara Lomzynska, Danielle Ferraresi, Michael Norton, David Austin, Chris P. Gale, Chris Wilkinson, Graham McClelland

**Affiliations:** University of Sunderland; North East Ambulance Service NHS Foundation Trust ORCID iD: https://orcid.org/0000-0003-3744-2710; University of Sunderland; North East Ambulance Service NHS Foundation Trust; North East Ambulance Service NHS Foundation Trust; North East Ambulance Service NHS Foundation Trust; North East Ambulance Service NHS Foundation Trust; South Tees NHS Foundation Trust; Newcastle University ORCID iD: https://orcid.org/0000-0003-4606-1055; University of Leeds; Leeds Teaching Hospitals NHS Trust ORCID iD: https://orcid.org/0000-0003-4732-382X; University of York; University of Leeds ORCID iD: https://orcid.org/0000-0003-0748-0150; Northumbria University ORCID iD: https://orcid.org/0000-0002-4502-5821

**Keywords:** AED, bystander, drone, EMS, paramedic

## Abstract

**Introduction:**

Out-of-hospital cardiac arrest (OHCA) is a leading cause of death in Europe. Early defibrillation is associated with improved outcomes. While this may be delivered by members of the public using an automated external defibrillator (AED), they are used infrequently. Drone delivery of an AED may enable quicker defibrillation compared to awaiting arrival of emergency medical services. Little is known about how members of the public may react to AED delivery or about the potential impact of retrieving an AED on the provision of high-quality cardiopulmonary resuscitation (CPR).

**Methods:**

A feasibility study using a simulated OHCA scenario was completed by members of the public. Participants performed CPR on a manikin, guided by an ambulance service call handler, which was interrupted by AED delivery. CPR quality and the duration of the interruption for AED retrieval were recorded, and participants’ feedback on the scenario was collected using a survey.

**Results:**

Twelve participants completed the study. Overall, a median of 61% (interquartile range [IQR] 21–79) of chest compressions were delivered at the correct speed, and 99% (IQR 78–100) at the correct depth. CPR was discontinued for a median of 116 (96–135) seconds to retrieve an AED and deliver a shock. Participants described the scenario as stressful and challenging, were supportive of the concept of AED delivery by drone and emphasised the value of call-handler instructions and guidance.

**Conclusion:**

This study demonstrated the feasibility of a process and outcomes evaluation of simulated drone-delivered AED to members of the public. The retrieval process was associated with notable interruption in the delivery of CPR, but it remains unknown whether any impact of this may be offset by expedited use of the AED. Understanding the likely overall impact of drone delivery of AEDs on patient outcomes is critical before this approach should be considered in clinical practice.

## Introduction

Out-of-hospital cardiac arrest (OHCA) is a leading cause of death in Europe (Gräsner et al., 2021). Where resuscitation is attempted, the average survival for OHCA to hospital discharge is around 8%; however, in shockable cardiac arrest cases this figure is more favourable, 20–30% in European countries (Gräsner et al., 2021). An automated external defibrillator (AED) may be used by members of the public, and is associated with improved survival in OHCA (Elhussain et al., 2023), yet in the UK an AED is used in fewer than one in 10 cases (Hawkes et al., 2017).

The limited uptake of AEDs in OHCA is multifactorial and relates to participant reluctance due to fear of causing harm or adverse consequences, or due to a lack of awareness (Smith et al., 2017). Access is also suboptimal, as the average retrieval distance from a person’s home to their nearest AED across Great Britain is over 1.4 kilometres (Burgoine et al., 2023). This distance, and the associated retrieval time, could potentially be overcome by delivering an AED directly to the location of an OHCA by drone. AED drone delivery has been evaluated in Sweden, where the AED arrived before an ambulance in 67% of cases of OHCA, with a median time advantage over the ambulance of 3 minutes and 14 seconds (Schierbeck et al., 2023).

While delivery of an AED by drone is technically possible, the impact of drone delivery on the provision and quality of bystander-provided cardiopulmonary resuscitation (CPR) and patient clinical outcomes is not known. Therefore, we conducted a feasibility study to explore the impact of drone delivery of an AED on CPR delivery in a simulated OHCA and report the participant experience of this.

## Methods

A feasibility study was carried out to evaluate lay participants’ responses to the delivery of an AED during a OHCA scenario where they were being guided by an ambulance call handler. Ethical approval was granted by the University of Sunderland (UoS) ethics board (Reference number: 027662).

### Participants

Volunteers were recruited from the UoS patient and carer public involvement group via a gatekeeper and internal advertisement. Participants were purposively selected to maximise the diversity of the sample in terms of age, sex and previous experience of CPR training. Participants were eligible to participate if they were over 18 years of age and were not a healthcare professional.

### Intervention

The scenario was designed to simulate a typical OHCA that happens in a home, and for practical reasons we assumed a single bystander was present who could phone for an ambulance. Participants were given a standard short briefing, explaining the scenario, which was of a close family member or friend being found collapsed at home, and the participant being the only available responder. The scenarios were conducted at UoS paramedic training facilities in August 2024. A Laerdal QCPR manikin was placed on the floor with clear space around, good lighting and a soft mat to kneel on; an AED (Laerdal AED Trainer 2) was placed out of sight of the participant through an external door 11 metres from the manikin, to simulate the distance from a room in a home to the street or garden where a drone may be able to deliver an AED (Supplementary 1). This distance was chosen for logistical reasons and to replicate similar experimental conditions as described by Sanfridsson et al. (2019), Schierbeck et al. (2022) and Smith et al. (2024). In practice, it has been found in one study that AED delivery by drone within 15 metres of a building or patient was completed in 91% of cases (Schierbeck et al., 2023).

The patient was in a shockable rhythm throughout the scenario. The scenario started with the participant being handed a mobile phone, which was connected to a North East Ambulance Service NHS Foundation Trust (NEAS) NHS Trust health advisor, who followed the NHS Pathways call-handler system script to instruct the participant on how to deliver CPR. After three minutes, the call handler informed the participant that an AED had been ‘delivered’ and was outside the building. This timing was selected to replicate current practice, where in a prospective cohort study of drone AED delivery, the median delivery time was 3 minutes and 17 seconds (Schierbeck et al., 2023). When the participant returned with the AED, the call handler used the NHS pathways cardiac arrest script with additional instructions to apply and use the AED, followed by further CPR. The scenario concluded after 7 minutes, at which point an ambulance was stated to have arrived.

### Data sources and measurement

CPR metrics were recorded automatically by the Laerdal QCPR manikin, including the proportion of compressions that were delivered at a rate of between 100 and 120 per minute, compressions per minute, the proportion that were delivered at a depth of between 5 and 6 cm and the proportion with correct release during the scenario.

Timings were noted by an observer who was in the scenario room and corroborated with video recordings. These included the time interval from the notification of AED delivery to returning to the manikin and delivering a shock. The time interval during which CPR was stopped (time ‘off chest’) from the AED notification is also reported. A short, structured debrief survey was administered by members of the research team post scenario to gather information on the participant’s experience (Supplementary 2).

### Analytical methods

Descriptive statistics were used to summarise the quantitative data, using medians and interquartile range for non-normally distributed data, means with standard deviation for normally distributed data, or proportions. Survey results are presented following simple content analysis (Bengtsson, 2016).

## Results

Twelve participants volunteered and completed the scenario; five were female (42%) and seven were male (58%) (Supplementary 3). All were educated to GCSE level or above, and half reported previous training in CPR.

All participants were able to follow the instructions to perform CPR and complete the simulation. The simulation results indicated that the time taken to collect the AED from notification ranged from 7 to 60 seconds, with a median time of 20 seconds (IQR 15‒20) (Table 1). The mean time to apply the AED after collection was 61 seconds (±19.9). The total time to deliver a shock from the notification of AED delivery ranged from 89 to 180 seconds, with a mean time of 112 seconds (±32.2). The duration without CPR, or total time off chest from AED notification, ranged from 77 to 156 seconds, with a mean of 110 seconds (±32.2).

**Table 1.** Simulation results.

Variable	Range (min to max)	Median with IQR (or mean with SD)	National benchmarking standards (Soar et al., 2021)
Time to collect AED from notification of AED delivery (seconds)	7–60	20 (15–23)	N/A
Time to apply AED from AED collection (seconds)	40–102	Mean (±SD) 61 (±19.9)	N/A
Total time taken to shock patient from notification of AED delivery (seconds)	89–180	Mean (±SD) 112 (±32.2)	N/A
Total time off chest from AED notification (i.e. time with no CPR being performed) (seconds)	77–156	116 (96–135)	No more than 10 seconds
Total compressions performed	329–508	394 (374–426)	N/A
Mean compression rate	90–116	Mean (±SD) 104.6 (7.9)	100–120 per minute
% of compressions performed at the correct speed	1–80%	61% (21–79%)	N/A
% of compressions performed at the correct depth	5–100%	99% (78–100%)	N/A
% of compressions performed with correct release (recoil)	45–100%	Mean (±SD) 95.3% (6.14%)	N/A

The mean compression rate was 104.6 (±7.9) per minute, with 61% of compressions delivered at the correct speed (ranging from 1% to 80%). Almost all compressions (99%) were delivered at the correct depth, while the average release (recoil) achieved was 95.3% (±6.14%).

### Qualitative data

Participants were interviewed about their experiences post scenario and their responses are summarised below.

#### Emotional and psychological reactions

- **Daunting/stressful experience:** All but two participants felt overwhelmed and anxious during the scenario. The two participants who did not have this experience had received prior CPR training, and described this within their interview as something that helped them stay calm.
  *The scenario was tiring, and the whole thing was stressful even with a manikin.* (Participant 2)
- **Support and guidance:** The participants highlighted the importance of emotional support from the call handler to help them through the scenario. All the participants commented that the call handler had a positive impact on their performance.
  *I felt supported by the coaching of the call handler.* (Participant 12)
- **Anxiety about leaving the patient:** Several participants had concerns about leaving a patient unattended to retrieve the AED. This was visually seen during the scenario in their behaviour, hesitating to leave the patient unattended.
  *I felt anxious leaving the patient, what if something happened to them, or if they died and nobody was with them?* (Participant 12)

#### Physical and cognitive challenges

- **Physical difficulty with CPR:** Multiple participants reported the difficulty of performing CPR. The participants commented on how this surprised them and caught them off guard.
  *Doing CPR was hard … it was tiring to do CPR for so long.* (Participant 10)
- **Uncertainty and confusion:** Participants expressed confusion about their actions during CPR, with concerns over CPR hand placement, depth and whether to ‘breathe’ for the patient.
  *I wanted to make sure I did it right, but I wasn’t sure.* (Participant 2)

#### Interaction with equipment and technology

- **Navigating the AED:** Using the AED was perceived as challenging by all the participants, especially under the stress of the scenario. Participants reported that they did not know if they were ‘allowed’ to use an AED, what to do with it and where to place the pads. The call handler was reported as a ‘helpful factor’ in the process of using the AED for many participants.
  *It was hard to use AED, the instructions were difficult.* (Participant 10)
- **Thoughts on drone delivery:** Participants were generally positive about drone delivery but expressed concerns about the clarity of instructions. No other concerns were noted.
  *The drone idea is great, it wouldn’t faze me, but the instructions need to be clear.* (Participant 2)

## Discussion

We have shown that studying bystander CPR in a scenario-based OHCA, augmented by AED delivery and involving lay participants is feasible. We showed that participants were able to follow instructions on CPR delivery and successfully retrieve an AED. However, retrieval of an AED from 11 metres and delivering defibrillation was associated with an almost two-minute interruption in CPR.

Our findings closely align to the results of a recently published drone-AED delivery simulation study conducted by Smith et al. (2024). Most notably, their study recorded a median ‘hands-off’ time of 109 seconds, compared to our 110 seconds finding. Other work has also demonstrated comparable interruptions in CPR when retrieving drone-delivered AEDs, though the drone in that case was positioned further away (12‒15 metres) (Starks et al., 2024). This interruption in compressions is likely to be clinically significant and decrease the probability of successful resuscitation (Catalisano et al., 2024; Soar et al., 2021), which must be weighed against the potential of earlier defibrillation where there is a sole responder. It is possible that drone-delivered AEDs are more appropriate in circumstances where there is more than one bystander.

Most participants achieved guideline-directed CPR depth and recoil, with a reasonable compression rate. Real-time coaching from the NEAS Trust call handler is likely to have contributed to this, which correlates with participants’ feedback. This is comparable with previous work (Smith et al., 2024; Starks et al., 2024) and adds to the existing literature describing the benefit of call-handler coaching (Nikolaou et al., 2019).

While drone-assisted AED delivery is being trialled in other countries, regulatory barriers in the UK currently restrict its widespread testing or use. However, alongside work on the technical delivery mechanisms, it is crucial to understand its impact on bystander behaviour (Finney et al., 2025) and the potential impact on patient outcomes and modelling to optimise drone-station placement. There are also challenges in the interface with existing emergency response systems, alongside the need for health economic evaluation.

This work is novel, topical and carefully conducted. However, we acknowledge limitations. The observer effect may have influenced participant performance, and remote observation might enhance realism in future simulations. The lack of perfusion data from the QCPR device and technical issues also limited the scope of CPR quality assessment. Future studies should aim to incorporate more advanced technology and live systems to improve both data quality and realism. Last, the scenario only simulated a shockable OHCA patient, and future studies must consider the patient who may present in a non-shockable OHCA rhythm.

## Conclusion

This study demonstrated the feasibility of evaluating the impact of drone-delivered AEDs in a simulated OHCA scenario and provides insights into the challenges and potential benefits of this technology. Notably, AED retrieval from a short distance was associated with significant interruptions in CPR, which could hinder clinical outcomes where there is a sole responder. These findings indicate that clinical trials reporting patient outcomes will ultimately be needed to prove the safety and efficacy of drone-delivered AEDs in routine practice.

## Acknowledgements

We are grateful to Dr Lesley Scott for recruiting participants, and to Mr Kenny Fox for providing technical services during the data collection.

## Author contributions

OF conceptualised the project. OF, GM, CW and KS designed the study. OF applied for ethical approval. OF, GM, CW and KS collected the data. OF analysed the data. OF, GM, CW and KS interpreted the results and OF, GM, CW and KS wrote the manuscript. All authors were involved in the review process and all authors approved the final draft. OF acts as the guarantor for this article.

## Conflict of interest

None declared.

## Funding

None.
